# Outcomes and costs of ureteroscopy, extracorporeal shockwave lithotripsy, and percutaneous nephrolithotomy for the treatment of urolithiasis: an analysis based on health insurance claims data in Germany

**DOI:** 10.1007/s00345-021-03903-2

**Published:** 2021-12-15

**Authors:** Claudia Schulz, Benedikt Becker, Christopher Netsch, Thomas R. W. Herrmann, Andreas J. Gross, Jens Westphal, Thomas Knoll, Hans-Helmut König

**Affiliations:** 1 Department of Health Economics and Health Services Research, University Medical Center Hamburg-Eppendorf, Martinistr. 52, 20246 Hamburg, Germany; 2 Department of Urology, Asklepios Hospital Barmbek, Hamburg, Germany; 3 Department of Urology, Spital Thurgau AG, Kantonsspital Frauenfeld, Frauenfeld, Switzerland; 4 Department of Urology and Pediatric Urology, Hospital Maria Hilf, Alexianer Krefeld GmbH, Krefeld, Germany; 5 Department of Urology, Klinikum Sindelfingen-Boeblingen, Sindelfingen, Germany

**Keywords:** Postoperative complications, Health care costs, Administrative claims, Healthcare, Sick leave, Urolithiasis

## Abstract

**Purpose:**

Comparisons of ureteroscopy (URS), extracorporeal shockwave lithotripsy (SWL), and percutaneous nephrolithotomy (PCNL) for urolithiasis considering long-term health and economic outcomes based on claims data are rare. Our aim was to analyze URS, SWL, and PCNL regarding complications within 30 days, re-intervention, healthcare costs, and sick leave days within 12 months, and to investigate inpatient and outpatient SWL treatment as the latter was introduced in Germany in 2011.

**Methods:**

This retrospective cohort study based on German health insurance claims data included 164,203 urolithiasis cases in 2008–2016. We investigated the number of complications within 30 days, as well as time to re-intervention, number of sick leave days and hospital and ambulatory health care costs within a 12-month follow-up period. We applied negative binomial, Cox proportional hazard, gamma and two-part models and adjusted for patient variables.

**Results:**

Compared to URS cases, SWL and PCNL had fewer 30-day complications, time to re-intervention within 12 months was decreased for SWL and PCNL, SWL and PCNL were correlated with a higher number of sick leave days, and SWL and particularly PCNL were associated with higher costs. SWL outpatients had fewer complications, re-interventions and lower costs than inpatients. This study was limited by the available information in claims data.

**Conclusion:**

URS cases showed benefits in terms of fewer re-interventions, fewer sick leave days, and lower healthcare costs. Only regarding complications, SWL was superior. This emphasizes URS as the most frequent treatment choice. Furthermore, SWL outpatients showed less costs, fewer complications, and re-interventions than inpatients.

**Supplementary Information:**

The online version contains supplementary material available at 10.1007/s00345-021-03903-2.

## Introduction

Urolithiasis is a common disease with an lifetime risk of 9% [1] and an recurrence rate of 30–50% within 10 years [2]. The prevalence and incidence have been increasing in the last decades [3, 4]. Risk factors are e.g., male sex, nutrition, lifestyle and physical activity [5], and comorbid conditions as obesity and diabetes mellitus [1, 6].

Due to its acute nature, urolithiasis requires a large volume of health care resources and generates high costs. In the US, annual direct health care costs summed up to approx. USD 4.5 billion [7]. Urolithiasis primarily affects working-age adults [8], thus, the economic burden includes productivity loss. Annual work loss was estimated to amount to 3.1 million workdays with associated indirect costs of USD 775 million in the US [7].

The three most common procedures for removing urinary tract stones are extracorporeal shockwave lithotripsy (SWL), ureteroscopy (URS), and percutaneous nephrolithotomy (PCNL). SWL rarely entails complications, but has some contraindications and a modest stone-free rate [9, 10]. URS has little contraindications and a sufficient stone-free rate [9]. URS seems to be more cost-effective than SWL due to better stone-free rates and lower costs [11–13]. There is a trend away from SWL and towards URS [14–16]. PCNL entails a high stone-free rate, but more complications and higher costs [9, 11].

In Germany, all three treatments usually take place on an inpatient basis. For this purpose, urolithiasis patients have to stay in hospital for 3.6 days on average [17], causing high health care costs. Yet, outpatient treatment may decrease the required health care resources and costs. In Germany, selected surgery can also be provided in an outpatient hospital setting by hospital physicians, if eligible. Additionally, there are office-based physicians, e.g., general practitioners or specialists, who provide diagnostics and non-surgical treatment. Since 2011, not only hospital physicians may provide outpatient surgery, but also office-based physicians, e.g. urologists. Based on a contract with a hospital, they may offer outpatient treatments for the hospital’s account, which simplified and significantly increased the conduction of eligible treatments such as outpatient SWL. If the costs for SWL decrease when conducted in the outpatient setting, this would improve the cost-effectiveness of SWL.

As urinary tract stones are likely to reoccur, outcomes such as stone-free rate, health care costs and sick leave days in the long run are highly relevant. Yet, existing literature mainly focused on a short follow-up [13, 15]. Therefore, our research objectives were to compare (1) the inpatient hospital treatments URS, SWL and PCNL, and (2) inpatient and outpatient hospital treatment options for SWL for urolithiasis cases regarding health-related and economic outcomes within 1 year.

## Methods

### Study design and data sources

For this retrospective cohort study, health insurance claims data were provided by the Scientific Institute of the AOK (“Wissenschaftliches Institut der AOK”) (WIdO). The WIdO administers the data of the largest association of statutory health insurance companies in Germany covering about one-third of the German population. Data were available for the years 2007–2017. We used 1 year preceding the index treatment as a washout period and 1 year as a follow-up period; thus, for research objective (1) the years 2008–2016 were used as index period to identify urolithiasis cases and their treatments. For research objective (2), we used the years 2011–2016 as index period, as outpatient hospital treatment with SWL was rare earlier. As we used anonymized claims data, informed consent of the study participants and approval of an ethics committee were not applicable.

### Inclusion and exclusion criteria

All cases of patients insured by the AOK, living in Germany, and with an incident urolithiasis treatment with URS, SWL or PCNL during the index period, both emergencies and elective cases, were included. We considered inpatient hospital treatment of URS, SWL and PCNL for research objective 1, and SWL inpatient and outpatient hospital treatment for research objective 2. Cases of urolithiasis treatment were identified using the hospital discharge diagnosis N20-N23 of the International Classification of Diseases, German Modification (ICD-10). The codes used for the identification of treatments can be found in Supplementary Table 1.

We excluded cases with no incident urolithiasis treatment, i.e. cases with URS, SWL or PCNL treatment in the 12-month period preceding the index treatment. We further excluded combined treatments within the same day (*n* = 1055), as we could not clearly assign the effects to one of the treatments, as well as outpatient hospital URS and PCNL treatments (*n* = 217) which both should be rare and unusual events.

### Definition of variables

The following variables were used as outcomes: Common treatment complications within 30 days were measured with specific ICD- and OPS-codes (Supplementary Table 1). Re-interventions as a proxy for the stone-free rate within 365 days were measured as additional treatment of URS, SWL or PCNL after index hospital discharge. Sick leave days within 365 days were recorded in claims data. Health care costs within 365 days were measured as total, inpatient and outpatient hospital, and ambulatory costs. They were reported in 2016 Euro and adjusted for inflation using the Gross Domestic Product price index [18, 19]. In Germany, health care costs in hospitals are calculated as a fixed amount based on diagnosis-related groups per inpatient or outpatient hospital case, i.e. one amount for all treatments during one hospital stay. Ambulatory costs are calculated as a fixed amount per treatment and then summed up per physician contact. Apart from negligible co-payments, health insurances reimburse these costs to health care providers. By using the corresponding claims data, we applied a payer perspective on costs. To avoid bias by extreme outliers, all costs were winsorized at the 99% percentile.

The following characteristic was used for risk adjustment: Sex, age at the index date, year of index treatment, and for research objective (1) index inpatient hospital length of stay. Additionally, Elixhauser comorbidities were defined based on the ICD-10 codes at index hospital admission [20, 21]. Assessment of stone-free status was observed within 12 weeks after surgery.

### Statistical analysis

We analyzed variables descriptively for the full sample, and compared them between (1) inpatient hospital URS, SWL and PCNL cases, and (2) between inpatient and outpatient hospital SWL cases, using numbers and share, or mean and standard deviation. Furthermore, for complications and sick leave days, we used generalized linear models with negative binomial distribution and log link function. For time to re-intervention, we used a Cox proportional hazard model [22]. We found no serious violations when checking the proportional hazards assumption visually [23] and by testing the interaction between time and covariates, and between time and Schoenfeld residuals [24]. For health care costs, we either used generalized linear models with gamma distribution and log link function, if costs occurred for all cases, or two-part models, if costs only occurred for some but not all cases. The two-part models consisted of a logistic regression to estimate the likelihood of costs occurring for a case, and a gamma regression to estimate the amount of costs, if occurring. Cost results were reported as average marginal effects.

## Results

### Comparison of inpatient URS, SWL and PCNL treatment

There were 164,203 inpatient urolithiasis cases. 104,923 (63.90%) were treated with URS, 45,773 (27.88%) with SWL and 13,507 (8.23%) with PCNL. The share of males was about two thirds for the URS and SWL group, and lower for the PCNL group. On average, patients were 52.28 years old, with PCNL patients slightly older. SWL cases were mostly treated on the day of hospital admission, while URS and PCNL cases were treated later. About three quarters of the urolithiasis cases received post-operative assessments of stone-free status, with the highest rates for SWL and lowest for URS cases.

SWL cases had fewer and PCNL cases slightly fewer 30-day complications than URS cases. Time to re-intervention within 12 months was shorter for SWL and PCNL compared to URS. SWL cases had more and URS cases fewer re-hospitalizations than PCNL cases. SWL and PCNL were associated with more sick leave days compared to URS. Regarding health care costs, SWL and particularly PCNL were costlier than URS. For PCNL, cost drivers were the index inpatient hospital stay and the total inpatient hospital costs (Table 1).

**Table 1:** Descriptive results for the comparison of URS, SWL and PCNL cases

	Total		URS			SWL			PCNL	
*N*, %	164,203		104,923	63.90%	45,773		27.88%	13,507		8.23%
Baseline characteristics (date of treatment)										
Sex: *n*, %										
Female	56,078	34.15%	34,039	32.44%	16,059		35.08%	5980		44.27%
Male	108,125	65.85%	70,884	67.56%	29,714		64.92%	7527		55.73%
Age: mean, SD	52.28	16.29	51.62	16.59	52.82		15.53	55.56		16.00
Time to surgery [days]: *n*, %										
0	59,583	36.29	30,877	29.43	25,201		55.06	3505		25.95
1	45,987	28.01	29,899	28.50	10,328		22.56	5760		42.64
2–3	21,636	13.18	14,292	13.62	6,292		13.75	1052		7.79
4 or more	36,997	22.53	29,855	28.45	3,952		8.63	3190		23.62
Length of stay [days]: mean, SD	6.80	7.91	7.19	8.23	4.85		6.15	10.46		8.83
Cases with post-operative assessments of stone-free status: *n*, %										
In total	119,092	72.53	70,350	67.05	37,733		82.44	11,009		81.51
Via ultrasound of retroperitoneal space, ureter and bladder	114,660	69.83	68,594	65.38	36,414		79.55	9652		71.46
Via kidney, ureter, and bladder radiography	9860	6.00	2619	2.50	3469		7.58	3772		27.93
Via abdominal computed tomography scan	26,590	16.19	14,283	13.61	8,982		19.62	3325		24.62
Follow-up within 30 days after treatment										
Number of complications: mean, SD	0.27	0.54	0.31	0.54	0.18		0.48	0.29		0.63
Follow-up within 365 days after treatment										
Number of re-interventions: *n*, %	0.61	1.27	0.29	0.83	1.34		1.68	0.67		1.40
Time to re-Intervention [days]: mean, SD	263.63	154.84	312.15	119.93	153.46		168.57	260.01		155.01
Number of re-hospitalizations: mean, SD	0.99	1.51	0.85	1.43	1.28		1.61	1.10		1.66
Number of sick leave days: mean, SD	16.88	40.88	16.45	40.31	17.72		41.56	17.35		42.81
Total health care costs [€]: mean, SD	6167	5564	5673	5423	6384		5281	9266		6458
Thereof due to inpatient hospital treatment	5412	5342	4954	5180	5552		5101	8500		6256
Thereof during index hospital stay	2731	1274	2578	949	2430		1320	4938		1210
Thereof due to outpatient hospital treatment	49	177	50	176	47		177	48		182
Thereof due to ambulatory treatment	705	712	669	705	785		724	717		707

*URS* Ureteroscopy, *SWL* Extracorporeal shockwave lithotripsy, *PCNL* Percutaneous nephrolithotripsy, *SD* Standard deviation

Multivariate regression results (Table 2) underpin the descriptive results. SWL (OR 0.67; *p* < 0.0001) and, to a lesser extent, PCNL cases (Odds ratio (OR) 0.89; *p* < 0.0001) were associated with significantly less complications than URS cases. SWL (Hazard ratio (HR) = 5.60; *p* < 0.0001) and PCNL (HR = 2.01; *p* < 0.0001) cases were significantly more likely to receive re-interventions than URS cases. The likelihood for sick leave days was significantly increased for PCNL (OR 1.21; *p* < 0.0001) and SWL (OR 1.16; *p* < 0.0001) cases compared to URS cases. Total health care costs were significantly increased for SWL (average marginal effect (AME) = 1033 €; *p* < 0.0001) and particularly for PCNL (AME = 2798 €; *p* < 0.0001) cases. Interestingly, the index inpatient costs were significantly lower for SWL (AME = − 45 €; *p* < 0.0001) than for URS cases. However, within the follow-up period, inpatient (AME = 918; *p* < 0.0001) and ambulatory (AME = 63; *p* < 0.0001) costs were significantly higher for SWL than for URS cases, which explains the higher total costs. For PCNL cases, particularly the index hospital costs (AME = 2145 €; *p* < 0.0001) and the total inpatient hospital costs (AME = 2814 €; *p* < 0.0001) were significantly higher than for the URS cases.

**Table 2:** Multivariate results for the comparison of URS, SWL and PCNL cases

	SWL			PCNL		
	OR/HR/AME	*p* value	95% CI	OR/HR/AME	*p* value	95% CI
30-day number of complications: OR	0.67	< 0.0001	0.65–0.69	0.89	< 0.0001	0.86–0.92
365-day time to re-intervention: HR	5.60	< 0.0001	5.49–5.71	2.01	< 0.0001	1.94–2.08
365-day number of sick leave days: OR	1.16	< 0.0001	1.13–1.2	1.21	< 0.0001	1.16–1.28
365-day total health care costs: AME [€]	1033	< 0.0001	1017–1050	2798	< 0.0001	2732–2865
Thereof due to inpatient hospital treatment	918	< 0.0001	902–934	2814	< 0.0001	2747–2882
Thereof during index hospital stay	− 45	< 0.0001	− 48 to 42	2145	< 0.0001	2122–2168
Thereof due to outpatient hospital treatment	6	< 0.0001	4–8	− 2	0.307	− 5 to 2
Thereof due to ambulatory treatment	63	< 0.0001	59–68	− 10	< 0.0001	− 17 to 4

All models were adjusted for sex, age, index year and Elixhauser comorbidities. Reference category for all models was ureteroscopy

*SWL* extracorporeal shockwave lithotripsy; *PCNL* percutaneous nephrolithotripsy; *OR* odds ratio; *HR* Hazard ratio; *AME* average marginal effect; *CI* confidence interval

### Comparison of inpatient and outpatient SWL treatment

We compared 28,358 inpatient and outpatient hospital SWL cases, of whom 26,227 (92.49%) were treated inpatient and 2131 (8.13%) outpatient. Both groups were similar in terms of sex and age. The inpatient group had considerably more complications and re- interventions, but fewer re-hospitalizations and sick leave days than the outpatient. Total health care costs and index treatment costs were higher for the inpatient than for the outpatient cases (Supplementary Table 2).

The likelihood for complications (OR 0.07; *p* < 0.0001) or re-interventions (HR = 0.92; *p* = 0.006) was significantly decreased for outpatient compared to inpatient SWL cases, whereas the likelihood for sick leave days was non-significantly increased (OR 1.06; *p* = 0.34). The outpatient SWL group was associated with significantly lower total health care costs (AME = − 942 €; *p* < 0.0001) than the inpatient SWL group. Inpatient hospital costs (AME = − 1447 €; *p* < 0.0001) and index treatment stay costs (AME = − 1911; *p* < 0.0001) were significantly lower and outpatient hospital costs significantly higher for the outpatient SWL group (AME = 674 €; *p* < 0.0001) (Supplementary Table 3).

## Discussion

When comparing inpatient URS, SWL and PCNL cases, URS showed favorable results in most outcomes. URS had fewer re-interventions, sick leave days and costs within 1 year than SWL and PCNL cases, but more 30-day complications. When comparing SWL and PCNL, SWL cases had fewer complications and less costs, but PCNL cases had fewer re-interventions. Regarding sick leave days, both had similar outcomes.

When comparing health-related outcomes, our results for URS, SWL and PCNL stand in line with literature [10]. Our results emphasize the trend away from SWL and towards URS [14, 15]. However, for PCNL cases we found a slightly worse time to and a larger number of re-interventions than for URS cases contradicting existing literature [9]. In terms of stone-free rates, PCNL is usually preferred over URS [25, 26]. Probably, in our study PCNL was conducted for cases with particularly large or complex stones as intended, and these cases needed re-intervention more often than URS cases due to the complex nature of urolithiasis. In the same vein, the complex diagnosis and treatment may explain the longer time to surgery for PCNL and URS cases. Furthermore, we had no information on the treatment site of the re-intervention. Thus, some cases may have been additional treatments on the other side. It should be noted that re-intervention rates do not perfectly measure stone-free rates, but serves as a proxy. Similarly, common literature shows a higher rate of complications for PCNL than for URS cases opposing our results. Probably, not all occurring complications were coded in the claims data. We could not apply the established Clavien-Dindo classification of complications [27, 28] as it exceeds the information available in claims data.

URS cases showed the lowest total health care costs, although the index hospital treatment was more expensive for URS than for SWL cases. This may be caused by the high re-intervention rate for SWL cases which entailed higher total inpatient costs. There is evidence that URS is more cost-effective than SWL with both a higher stone-free rate and lower costs [11–13], although another study demonstrated lower costs for SWL [29]. PCNL cases had considerably higher costs, both during the index hospital stay and in total. The question arises if the higher costs can be justified by better stone-free rates reported in the literature [25, 26]. Two studies compared PCNL with SWL [30] and URS [31], and both showed higher costs and effectiveness for PCNL. Overall, the indications for the 3 treatment options differ, which limits the feasibility of health-economic evaluation.

Outpatient SWL cases had fewer complications, fewer re-interventions, and considerably less costs, both for the index treatment and overall 1-year costs than inpatient cases. However, based on these data, we cannot answer the question whether SWL outpatients have more favorable outcomes due to the outpatient treatment or due to selection effects. SWL may have been conducted in the outpatient setting only if cases had e.g., less comorbidities and a less complex urolithiasis. Yet, for eligible patients, this seems to be a viable and less costly option.

Our study has some limitations. Although we adjusted for various patient factors affecting the observed outcomes, they may not fully capture the patients’ risk profile. There are further risk factors, for example lifestyle or physical activity [5]. However, data on patient characteristics are only available to a limited degree in claims data. The quality of claims data may be limited, although they have a high validity due to their reimbursement purpose. Thus, the measurement based on claims data may not fully capture all e.g., complications. Furthermore, patients may be selected for a treatment due to certain factors which cannot be observed in claims data, e.g., stone size. Therefore, we cannot rule out a selection bias. Thus, our results are not causal effects of the treatment, but rather correlations for patients for whom the treatment was chosen.

Yet, our study adds to existing knowledge on the treatment of urolithiasis by drawing on a large and rich data set of more than 160,000 cases over 9 years. We used claims data which are less vulnerable to information bias, an issue common for survey data. The AOK has a high national coverage of about one-third of the German population, which makes our results fairly representative. To the best of our knowledge, this is the first comprehensive study comparing URS, SWL and PCNL for urolithiasis patients based on claims data.

## Conclusion

This study provides insights into outcomes and costs for urolithiasis treatments. Cases treated with URS showed benefits regarding long time without re-interventions, few sick leave days and low health care costs. This emphasizes URS as the most frequent treatment choice. SWL cases had fewer complications than URS cases. PCNL cases had the highest number of complications and health care costs. SWL and PCNL may be performed for cases with according indications [32]. Furthermore, SWL outpatients had less costs, fewer complications and re-interventions, and may therefore be a sufficient alternative for inpatient SWL treatment, if eligible.

## Supplementary Information

Below is the link to the electronic supplementary material.

Supplementary file1 (PDF 490 KB)

## Data Availability

The datasets supporting the conclusions of this article are owned by the German statutory health insurance AOK. Since public deposition of the data would breach ethical and legal compliance, data are only available upon formal request from the research institute of the AOK (WIdO). To request the data please contact the institutional body of the WIdO (wido@wido.bv.aok.de). To fulfill the legal requirements to obtain that kind of data, researchers must obtain a permission for a specific research question from the German Federal (Social) Insurance Office. Additionally, researchers must conclude a contract with the statutory health insurance regarding data access which can be requested from the “AOK-Bundesverband GbR” (Federal Association of Local Health Insurance Funds) under http://aok-bv.de/kontakt/. The licensee is permitted to use the data for the purpose of the research proposal within their company, exclusively. Thereby, a company is defined as an economical unit. Licensees are not allowed to pass the data to a third party, or to create Software or databases with the exception of scientific publications. Moreover, the study has to be approved by the data protection officer both at the statutory health insurance and the research institute.

## Code availability

Code is available by request. Please contact the corresponding author.
