# Supplementary material for: Outcomes and costs of ureteroscopy, extracorporeal shockwave lithotripsy, and percutaneous nephrolithotomy for the treatment of urolithiasis: an analysis based on health insurance claims data in Germany
Source: World J Urol. 2021 Dec 15;40(3):781–8. doi: 10.1007/s00345-021-03903-2 (PMC8948106; doi:10.1007/s00345-021-03903-2)
Supplement: Supplementary file 1 — Supplementary file1 (PDF 490 KB) [file 345_2021_3903_MOESM1_ESM.pdf]

Outcomes and costs of ureteroscopy, extracorporeal shockwave lithotripsy, and percutaneous nephrolithotomy for the treatment of urolithiasis: An analysis based on health insurance claims data in Germany

World Journal of Urology

Claudia Schulz [a]; Benedikt Becker [b]; Christopher Netsch [b]; Thomas R. W. Herrmann [c]; Andreas J. Gross [b]; Jens Westphal [d], Thomas Knoll [e], HansHelmut König [a]

[a] Department of Health Economics and Health Services Research, University Medical Center Hamburg-Eppendorf, Hamburg, Germany

[b] Department of Urology, Asklepios Hospital Barmbek, Hamburg, Germany

[c] Department of Urology, Spital Thurgau AG, Kantonsspital Frauenfeld, Frauenfeld, Switzerland

[d] Department of Urology and Pediatric Urology, Hospital Maria Hilf, Alexianer Krefeld GmbH, Krefeld, Germany

[e] Department of Urology, Klinikum Sindelfingen-Boeblingen, Germany

Corresponding author: Claudia Schulz, University Medical Center Hamburg-Eppendorf, Department of Health Economics and Health Services Research, Martinistr. 52, 20246 Hamburg, Germany, Phone +49 40 7410 54480, Fax +49 40 7410 40261, Email c.schulz@uke.de, ORCID: 0000-

Supplementary table 1: ICD- and OPS-codes for urolithiasis treatment, complications or assessments during or after treatment

**Diagnosis or treatment**	**ICD- or OPS-Codes**
Diagnosis	
Urolithiasis	N20, N21, N22, N23
Treatment	
Ureteroscopy	5-562.4, 5-562.5, 5-562.8, 5-550.21, 5-550.31
Extracorporeal shock wave lithotripsy	8-110.1, 8-110.2
Percutaneous nephrolithotomy	5-550.20, 5-550.30, 5-562.6, 5-562.7
Complications	
Embolization by angiography	3-612.1, 8-836.0a, 8-836.0q, 8-83b.3x, 8-836.ma, 8-836.mh
Surgical removal of urinary bladder tamponade	5-570.4
Placement of double-J catheter	8-137.00
Change of double-J catheter	8-137.10, 8-137.11
Ventilation	8-713
Blood transfusion	8-800.0, 8-800.1
	A49.0, A49.1, A49.3, A49.8, A49.9 A41.0, A41.1, A41.2, A41.4, A41.51, A41.52, A41.58, A41.8, A41.9 Sepsis
Bleeding and hematoma during or after treatment	T81.0
Shock during or after treatment	T81.1
Shock and sepsis during or after treatment	R57.2
Infection after treatment	T81.4, N39, N10
Vessel-related complications after treatment	T81.7

Unspecific complications after treatment	T81.9
Fever	R50.2, R50.8, R50.88, R50.9
Urinoma	R39.0
Post-operative assessment of stone-free status	
Ultrasound of retroperitoneal space, ureters, and bladder	In hospitals: 3-030, 3-05a, 3-05b; ambulatory: 33042, 33043, 33044
Kidneys, ureters, and bladder radiograph	In hospitals: 3-13d.0, 3-13d.5, 3-13d.6
Abdominal computed tomography scan	In hospitals: 3-207, 3-225; ambulatory: 34341

Abbreviations: ICD = International Classification of Diseases; OPS = operation and procedure codes.

Supplementary table 2: Descriptive results for the comparison of inpatient and outpatient cases of extracorporeal shockwave lithotripsy

	Total	Inpatient	Outpatient
N, %	28,358	26,227 92.49%	2,131 8.13%
**Baseline characteristics (date of treatment)**			
Sex: n, %			
… female	9,915 34.96%	9,173 34.98%	742 34.82%
… male	18,443 65.04%	17,054 65.02%	1,389 65.18%
Age: mean, SD	52.34 15.52	52.47 15.58	50.75 14.75
**Follow-up within 30 days after treatment**			
Number of complications: mean, SD	0.20 0.52	0.22 0.54	0.01 0.12
**Follow-up within 365 days after treatment**			
Number of re-interventions: n, %	1.30 1.57	1.32 1.59	1.13 1.20
Time to re-Intervention [days]: mean, SD	150.94 166.63	150.04 167.49	162.00 155.24
Number of re-hospitalizations: mean, SD	1.29 1.58	1.28 1.59	1.43 1.51
Number of sick leave days: mean, SD	19.31 43.23	19.14 43.20	21.35 43.64
Total health care costs [€]: mean, SD	6,443 5,279	6,555 5,310	5,062 4,653
… thereof due to inpatient hospital treatment	5,570 5,109	5,734 5,124	3,549 4,443
… thereof during index hospital stay	2,358 1,480	2,550 1,371	
… thereof due to outpatient hospital treatment	121 305	64 208	826 410

… thereof during index outpatient hospital treatment	46 166		611 150
… thereof due to ambulatory treatment	753 722	758 727	687 644

Abbreviations: SD = Standard deviation.

Supplementary table 3: Multivariate results for the comparison of inpatient and outpatient cases of extracorporeal shockwave lithotripsy

	Outpatient hospital treatment (ref: inpatient hospital treatment)		
	**OR/HR/AME**	**p-value**	**95% CI**
30-day number of complications: OR	0.07	<.0001	0.05-0.1
365-day time to re-intervention: HR	0.92	0.006	0.87-0.98
365-day number of sick leave days: OR	1.06	0.34	0.94-1.20
365-day total health care costs: AME [€]	-942	<.0001	-1,044--835
… thereof due to inpatient hospital treatment	-1,447	<.0001	-1,812--1,081
… thereof due to outpatient hospital treatment	674	<.0001	612-736
… thereof due to index treatment stay (inpatient or outpatient)	-1,911	<.0001	-1,909--1,913
… thereof due to ambulatory treatment	0	0.983	-16-16

All models were adjusted for sex, age, index year and Elixhauser comorbidities. Abbreviations: Ref = Reference category; OR = Odds ratio; HR = Hazard ratio; AME = Average marginal effect; CI = Confidence interval.
